# Keratouveitis caused by *Euphorbia* plant sap

**DOI:** 10.4103/0301-4738.53060

**Published:** 2009

**Authors:** Samar K Basak, Partho K Bakshi, Sabitabrata Basu, Soham Basak

**Affiliations:** Disha Eye Hospitals and Research Centre, Barrackpore, Kolkata - 700 120, West Bengal, India; 1 Kasturba Medical College, Manipal, Udupi - 576 104, India

**Keywords:** *Euphorbia* sap, keratitis, uveitis

## Abstract

The milky sap or latex of *Euphorbia* plant is highly toxic and an irritant to the skin and eye. This report illustrates the spectrum of ocular inflammation caused by accidental inoculation of latex of *Euphorbia* plant. Three patients presented with accidental ocular exposure to the milky sap of *Euphorbia* species of recent onset. The initial symptoms in all cases were severe burning sensation with blurring of vision. Visual acuity reduced from 20/60 to counting fingers. Clinical findings varied from kerato-conjunctivitis, mild to severe corneal edema, epithelial defects, anterior uveitis and secondary elevated intraocular pressure. All symptoms and signs had resolved by 10-14 days with active supportive medication. People who handle *Euphorbia* plants should wear eye protection. It is always advisable to ask the patient to bring a sample of the plant for identification.

The *Euphorbiaceae* family includes trees, succulents and herbaceous plants.[1] Different species of *Euphorbia* grow all over the world, either wild, or as cultivated specimens in the house or garden. The milky latex or sap is toxic and may cause intense inflammation of the skin and the eye.[2] Ocular toxic reaction varies from mild conjunctivitis to severe kerato-uveitis. There are a few case reports of permanent blindness resulting from accidental inoculation of *Euphorbia* sap into the eye.[2–4] Corneal involvement generally follows a typical sequence with worsening of edema with epithelial sloughing on the second day.[3,5] It is believed that some species are more toxic than the others.[6] When treated early and managed meticulously, the inflammation generally resolves without sequelae. Here, we present three cases of ocular toxicity caused by three different species of *Euphorbia*, namely, *E. trigona* (African milk tree)*, E. neriifolia* (Indian Spurge tree) and *E. milii* (Crown-of-thorns houseplant).

## Case Reports

### Case 1

**Figure 1:**
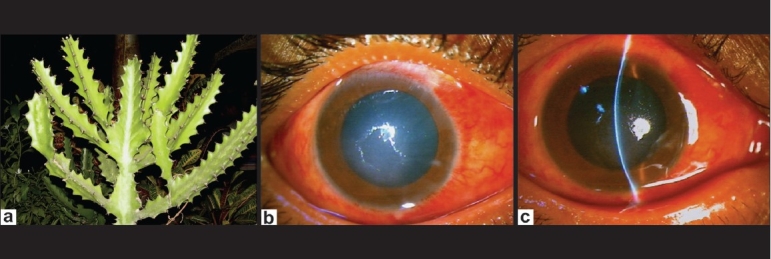
(a) Photograph of *Euphorbia trigona* plant (African milk tree); (b and c) Corneal epithelial defect, edema, stromal edema and moderate anterior uveitis – one day after exposure to *E. trigona* sap

A 60-year-old male was trimming his garden hedge *E. trigona* plant (African milk tree) [Fig. 1a] and got sprayed with milky sap into his right eye (RE). He had an immediate burning sensation and pain which was relieved partially by irrigation with water. After 16 h, he presented to us with pain, burning with gross dimness of vision in the RE. Best corrected visual acuity (BCVA) was 20/200 in the RE and 20/20 in the left eye (LE). On examination of the RE, there was mild lid edema and moderate conjunctival congestion and chemosis. There was loss of corneal epithelium and stromal edema. There was moderate anterior chamber reaction with 2+ cells and 2+ flare [Figs. 1b and c]. Intraocular pressure (IOP) was higher digitally. Fundus details appeared normal. The LE was essentially normal.

The eye was once again irrigated copiously with normal saline and treated with gatifloxacin (0.3%) eye drops four times daily, prednisolone acetate (1%) eye drops four times daily, homatropine (2%) eye drops three times daily, preservative-free (on surface) tears substitute and timolol maleate (0.5%) eye drops twice daily. The patient was observed closely as an outpatient. The corneal epithelium gradually healed over four days, and by 10 days all signs and symptoms had resolved and patient regained 20/20 vision.

### Case 2

**Figure 2:**
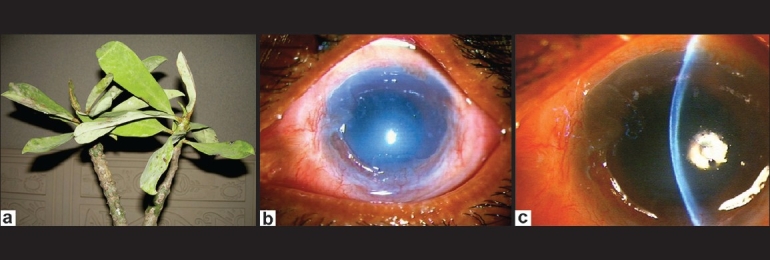
(a) Parts of *Euphorbia neriifolia* plant (Indian Spurge tree); (b and c) Large corneal epithelial lesion with stromal edema and mild anterior uveitis – two days after exposure to *E. neriifolia* sap

A 51-year-old man was pruning his overgrown species of *E. neriifolia* plant (Indian Spurge tree) [Fig. 2a] in his garden when he felt some sap enter into his LE. The eye became irritable and was immediately irrigated with tap water.

He presented four hours later. On examination, BCVA in the RE was 20/20 and in the LE 20/40. There was conjunctival hyperemia, moderate corneal edema and mild anterior chamber reaction. He had also a healed corneal pannus. The IOP was 13 mm Hg. The next day, the visual acuity had reduced to counting fingers at 1 meter. There was moderate lid edema and conjunctival congestion. Slit-lamp examination revealed a large corneal epithelial defect and moderate stromal edema [Figures 2b and c]. The IOP was higher digitally. The RE was unaffected and within normal limits. The patient was treated and followed up similar to Case 1. The corneal epithelium completely healed by the seventh day. By two weeks all signs and symptoms were resolved and the patient regained full vision.

### Case 3

**Figure 3:**
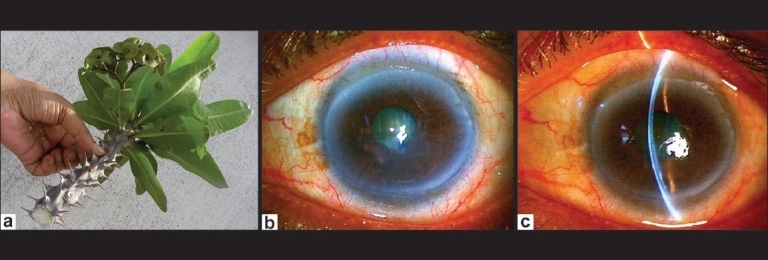
(a) Parts of *Euphorbia milii* houseplant (crown-of-thorns); white milky sap visible at the cut end; (b and c) Punctate epithelial lesions, corneal edema, Descemet's folds and moderate anterior uveitis – one day after exposure to *E. milii* sap

A 54-year-old woman was pruning her *Euphorbia milii* (crown-of-thorns) houseplant [Fig. 3a] when she felt a stinging sensation as a drop of sap entered her LE. She did not wash her eyes immediately. Fifteen minutes later she felt severe pain, blepharospasm and dimness of vision in the LE.

She presented three hours later and irrigation was done immediately with copious Ringer's lactate solution. On examination, BCVA in the RE was 20/20 and in the LE 20/120. There was conjunctival hyperemia, punctate localized corneal epithelial lesions, and moderate corneal edema with Descemet's folds. There was anterior uveitis with moderate anterior chamber reaction with 2+ cells and 2+ flare [Figures 3b and c]. The IOP was 14 mm Hg. The RE was within normal limits. The patient was treated and followed up similar to Case 1, except for timolol eye drops. The punctate epitheliopathy had completely resolved by Day three. By Day 15 all signs and symptoms had resolved and the patient regained BCVA of 20/20.

## Discussion

*Euphorbia* is a diverse plant genus consisting of more than 2000 species with worldwide distribution, chiefly in subtropical and temperate regions. Some species have thick succulent stems and are spiny, closely resembling cacti. They are distinguishably different by their peculiar flower and milky latex that contains irritant and carcinogenic diterpine esters.[7–9]

Though there are few case reports in literature, it is apparent from them that ocular changes follow a typical course, and the severity of the ocular inflammation may vary with the species of the plant.[6] Symptoms usually start immediately on contact with the milky latex. There is burning sensation, pain, photophobia and lacrimation which may worsen over hours even after copious irrigation. At first, there is mild diminution of vision, but may diminish further to 20/200 or counting fingers to hand movements within 24 h as Case 2 in this report. On initial examination, the corneal epithelium may be intact or with mild punctate epitheliopathy, but eventually it may show frank epithelial defect on the next day.[10] It takes around four to seven days for the epithelium to heal completely. There is stromal edema with Descemet's fold which decreases with time. The degree of anterior uveitis is variable and is particularly marked with certain species as in Case 1 and Case 3 in this report.[3] The degree of ocular inflammation may also vary with the amount of sap that enters the eye. Neglected cases can progress to blindness due to corneal scarring, complicated uveitis, and anterior staphyloma.[3,4]

The species of *Euphorbia* causing ocular toxicity reported earlier were mostly with *E. royaleana, E. lathyris* and *E. tirucalli.[*4,5,11,12] Only one case of ocular toxicity with *E. trigona* was reported earlier by Scott *et al.*[5] and they reported only corneal epithelial defect without edema and anterior chamber reaction. But in our Case 1, there was gross corneal edema with moderate anterior uveitis and secondary elevated IOP. This was possibly due to a greater amount of sap entering into the RE in our case. There was only one case report on *E. milii* by Eke *et al.*[6] and the patient presented with corneal epithelial defect and edema with mild anterior uveitis which was similar to our third case. To the best of our knowledge which includes MEDLINE search, we could not find any case report of ocular toxicity by the sap of *E. neriifolia* (Indian Spurge tree). If the patient presents early within 24 h, the treatment is antibiotic eye drops, topical corticosteroids, cycloplegics, tears substitute and IOP-lowering medications if necessary. No patching is required. With appropriate supportive therapy and close daily observation, the condition generally resolves completely within 10-15 days. In case of suspected bacterial infection and in the presence of a hypopyon, topical corticosteroids may be started later once the epithelial defect gets healed.[10]

In conclusion, the clinical course may be affected by particular species of *Euphorbia*, the amount of sap exposure, the time between exposure and irrigation, and host factors. Ophthalmologists managing *Euphorbia* keratouveitis should warn the patient that vision may get worse on the next day before it improves. It is always advisable to ask the patient to bring a sample of the plant for identification. People who work with *Euphorbia* species should wear protective goggles while handling the plant.
